# RB1CC1 Together with RB1 and p53 Predicts Long-Term Survival in Japanese Breast Cancer Patients

**DOI:** 10.1371/journal.pone.0015737

**Published:** 2010-12-22

**Authors:** Tokuhiro Chano, Kaichiro Ikebuchi, Yasuhiko Tomita, Yufen Jin, Hideo Inaji, Makoto Ishitobi, Koji Teramoto, Yasuko Ochi, Hitosuke Tameno, Ichiro Nishimura, Kahori Minami, Hirokazu Inoue, Takahiro Isono, Masao Saitoh, Taketoshi Shimada, Yasuo Hisa, Hidetoshi Okabe

**Affiliations:** 1 Department of Clinical Laboratory Medicine, Shiga University of Medical Science, Otsu, Japan; 2 Department of Otolaryngology-Head and Neck Surgery, Kyoto Prefectural University of Medicine, Kyoto, Japan; 3 Department of Pathology, Osaka Medical Center for Cancer and Cardiovascular Diseases, Osaka, Japan; 4 Department of Breast and Endocrine Surgery, Osaka Medical Center for Cancer and Cardiovascular Diseases, Osaka, Japan; 5 Department of Thoracic Surgery, Shiga University of Medical Science, Otsu, Japan; 6 Department of Microbiology, Shiga University of Medical Science, Otsu, Japan; 7 Department Central Research Laboratory, Frontier Research on Molecular Destruction and Reconstruction of Tooth and Bone, Tokyo Medical and Dental University, Tokyo, Japan; 8 Department of Cellular Physiological Chemistry, Frontier Research on Molecular Destruction and Reconstruction of Tooth and Bone, Tokyo Medical and Dental University, Tokyo, Japan; Bauer Research Foundation, United States of America

## Abstract

*RB1*-inducible coiled-coil 1 (RB1CC1) plays a significant role in the enhancement of the retinoblastoma tumor suppressor (RB1) pathway and is involved in breast cancer development. However, RB1CC1's role in clinical progression of breast cancer has not yet been evaluated, so, as a first step, it is necessary to establish its usefulness as a tool to evaluate breast cancer patients. In this report, we have analyzed the correlation between abnormalities in the RB1CC1 pathway and long-term prognosis, because disease-specific death in later periods (>5 years) of the disease is a serious problem in breast cancer. Breast cancer tissues from a large cohort in Japan were evaluated by conventional immunohistochemical methods for the presence of the molecules involved in the RB1CC1 pathway, including RB1CC1, RB1, p53, and other well-known prognostic markers for breast cancer, such as estrogen receptor, progesterone receptor, and human epidermal growth factor receptor 2. The correlation between the immunohistochemical results and clinical outcomes of 323 breast cancer patients was analyzed using a Kaplan-Meier log-rank test and a multivariate Cox proportional hazards regression analysis. Absence of nuclear RB1CC1 expression was associated with the worst prognosis (Log-rank test, Chi-Square value = 17.462, p<0.0001). Dysfunction of either one of RB1CC1, RB1, or p53 was associated with the highest risk for cancer-specific death, especially related to survival lasting more than 5 years (multivariate Cox proportional hazard ratio = 3.951, 95% Confidence Interval = 1.566–9.967, p = 0.0036). Our present data demonstrate that the combined evaluation of RB1CC1, RB1 and p53 by conventional immunohistochemical analysis provides an accurate prediction of the long-term prognoses of breast cancer patients, which can be carried out as a routine clinical examination.

## Introduction

Conventional prognostic markers of breast cancer, such as age, tumor-node-metastasis (TNM) stage, and hormone receptor status are lacking in their ability to predict the recurrence or disease-specific death in later periods (>5 years) of the disease, which is one of the greatest problems during the postoperative clinical follow-up[1]. Clinical assays for estrogen receptor (ER), progesterone receptor (PR) and human epidermal growth factor receptor 2 (HER2) are useful for choosing the best postoperative adjuvant therapy. The likelihood of distant recurrence and cancer-specific death in triple-negative [ER(−), PR(−) and HER2(−)] breast cancer is larger than that of non-triple-negative cases, and the maximum difference is observed at 3 years after diagnosis. Thereafter, the difference between these two groups decreases year by year up to 10 years[1], [2], [3]. Therefore, the development of other diagnostic parameters for the risk of death from cancer in later periods (>5 years) of the disease is a matter of great interest.

As in many other cancers, the prognosis of breast cancer seems to be intimately related to its cytogenetic disorders. The retinoblastoma tumor suppressor (RB1) protein regulates G1/S-phase cell cycle progression and is a critical mediator of antiproliferative signaling. RB1 has been reported to be aberrant in approximately 20% of breast cancer cases[4], [5], and to be associated with a poor disease outcome[5], [6]. However, the regulatory mechanism of RB1 has not been fully clarified yet, although its function has been shown to be regulated mainly by phosphorylation[7]. RB1 status has only infrequently been applied to breast cancer prognostication[6]. RB1-inducible coiled-coil 1 (RB1CC1: the symbol used here is approved by the Human Genome Organization [HUGO] Gene Nomenclature Committee; it is also known as FIP200, [focal adhesion kinase family-interacting protein of 200 kDa]) was identified as an RB1 pathway regulator that in particular enhances *RB1* transcription[8], [9]. A genetic rearrangement of *RB1CC1* has also been suggested to be involved in the tumorigenesis of breast cancer[9], [10]. In addition, RB1CC1 has been reported to be involved in proliferation[8], [11], growth[12], [13], apoptosis[14], [15] and autophagy[16], [17], [18]. Recently, we have demonstrated that nuclear RB1CC1 binds to the 201bp upstream GC-rich region (from the initiation ATG) of the *RB1* promoter and activates RB1 expression[19]. We have also reported that RB1CC1 forms a complex between p53 and/or hSNF5 (also known as BAF47 or INI1), acting as a chromatin-remodeling factor in cell nuclei, and that the complex provides a strong activation of *RB1, p16* and *p21* promoters[20]. The coordinated expressions of RB1, p16 and p21 influence the proliferation activity in clinical breast cancer. Therefore, the immunohistochemical status of RB1, p53 and RB1CC1 may predict tumor progression and the clinical prognosis of breast cancer patients[20].

Our present study is designed to establish a convenient routine clinical method to evaluate the influence of abnormalities in this newly established pathway—i.e. the RB1CC1, p53- RB1 pathway—on the long-term prognosis of breast cancer.

## Results

### Loss of nuclear RB1CC1 expression correlates with triple-negative phenotype of breast cancer

The correlation between nuclear RB1CC1 expression and other clinical parameters of the expanded cohort was analyzed statistically (Table 1). Loss of nuclear RB1CC1 [RB1CC1(−)] correlated significantly with negative PR expression (p = 0.0003) and with a triple-negative [ER(−), PR(−) and HER2(−)] phenotype of breast cancer (p = 0.0003). The use of chemotherapy was significantly higher in RB1CC1(−) patients than in RB1CC1(+) patients (p<0.0001), and was also higher in patients with triple-negative cancers than in those with non-triple-negative disease (Chi-square and Fisher's exact test, p<0.0001; data not shown).

**Table 1:** Patient and tumor characteristics stratified by nuclear RB1CC1 expression.

Feature	Number	Nuclear RB1CC1				*p-*Value
		negative	(%)	positive	(%)	
Menopause						0.1204
Pre-	142	37	(37)	105	(47)	
Post-	182	62	(62)	120	(53)	
anti-Estrogen therapy						0.0540
none	85	33	(33)	52	(23)	
performed	239	66	(67)	173	(77)	
**Chemotherapy**						**<0.0001**
none	179	33	(33)	146	(65)	
performed	145	66	(67)	79	(35)	
Radiation						0.1248
none	108	27	(27)	81	(36)	
performed	216	72	(73)	144	(64)	
T grade: tumor size						0.0805
≤T1	138	35	(35)	103	(46)	
T2≤	186	64	(65)	122	(54)	
Nodes						0.0567
negative	210	56	(58)	154	(69)	
positive	111	41	(43)	70	(31)	
Stage: TNM class						0.2605
≤IIA	228	64	(67)	164	(73)	
IIB≤	93	32	(33)	61	(27)	
ER						0.0689
positive	188	50	(51)	138	(61)	
negative	136	49	(49)	87	(39)	
**PR**						**0.0003**
positive	170	37	(37)	133	(59)	
negative	154	62	(63)	92	(41)	
HER2						0.1553
negative	290	85	(86)	205	(91)	
positive	34	14	(14)	20	(9)	
**Triple Negative**						**0.0003**
others	237	59	(60)	178	(79)	
triple negative	87	40	(40)	47	(21)	
p53						0.3200
normal	244	71	(72)	173	(77)	
abnormal	80	28	(28)	52	(23)	
RB1						0.0833
positive	308	91	(92)	217	(96)	
negative	16	8	(8)	8	(4)	
RB1CC1						------
positive	225	0	(0)	225	(100)	
negative	99	99	(100)	0	(0)	
RB1CC1/RB1/p53						------
normal	169	0	(0)	169	(75)	
abnormal	155	99	(100)	56	(25)	

Chi-square and Fisher's exact tests was used to evaluate the relationships between clinical parameters and nuclear RB1CC1 expression. p-value <0.05, statistically significant.

### RB1CC1 is a prognostic predictor in breast cancer patients

**Figure 1:**
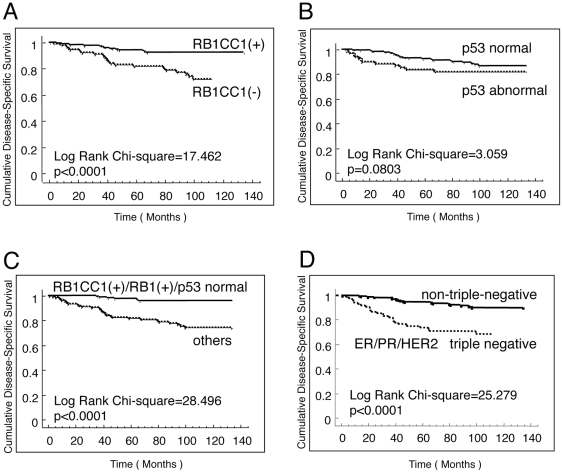
RB1CC1 is a novel prognostic factor in human breast cancer. Three hundred twenty-three cases of breast cancer were immunohistochemically evaluated and statistically analyzed relative to the clinical outcomes. Kaplan-Meier survival curves with Log-rank tests were performed for DSS evaluation of (**A**) RB1CC1, (**B**) p53, (**C**) the combination of RB1CC1/RB1/p53, and (**D**) triple-negative for ER/PR/HER2.

The Kaplan-Meier curve together with a log-rank analysis showed a significant relationship between nuclear RB1CC1 expression and breast cancer-specific survival (disease-specific survival: DSS), in which RB1CC1(−) predicted a worse prognosis for patients than did RB1CC1(+) (Chi-Square value = 17.462, p<0.0001; Fig. 1A). The relative hazards for DSS associated with 15 categorical risk factors were evaluated individually by a Cox proportional hazards analysis (Table 2). RB1CC1 conferred a significant relative hazard (p<0.0001) in addition to the risks of chemotherapy, tumor size, lymph node status, TNM class, ER, PR, triple-negative cancer, and RB1. Dysfunction of RB1CC1, RB1 or p53 (abnormal RB1CC1/RB1/p53) had the highest hazard ratio for DSS (Hazard ratio = 7.385, 95% Confidence Interval = 3.116–6.185, p<0.0001; Table 2). We reported earlier that nuclear RB1CC1 expression was highly correlated with expressions of RB1[19] and p16[19], [20], and that RB1CC1 and p53 provided a good stimulation of the coordinated expressions of RB1, p16 and p21, which, in turn, influenced tumor progression. Therefore, the immunohistochemical status of RB1, p53 and RB1CC1 might predict the prognosis of clinical breast cancer[20]. Indeed, together with the preliminary data of a small cohort (Fig. S1), these data suggested that the combined evaluation of RB1, RB1CC1, and p53 might provide useful information as prognostic biomarkers.

**Table 2:** Relative hazards of risk factors for breast cancer-specific death.

Features	Number	(%)	hazard ratio	(95% CI)	p-Value
Menopause					
Pre-	142	(44)	1.000		
Post-	181	(56)	0.871	(0.479–1.584)	0.6506
anti-Estrogen therapy					
none	85	(26)	1.000		
performed	238	(74)	0.920	(0.472–1.792)	0.8063
**Chemotherapy**					
**none**	**179**	**(55)**	**1.000**		
**performed**	**144**	**(45)**	**5.000**	**(2.398**–**10.418)**	**<0.0001**
Radiation					
none	108	(33)	1.000		
performed	215	(67)	0.571	(0.313–1.044)	0.0685
**T grade: tumor size**					
**≤T1**	**138**	**(43)**	**1.000**		
**T2≤**	**185**	**(57)**	**4.167**	**(1.855**–**9.346)**	**0.0005**
**Nodes**					
**negative**	**210**	**(66)**	**1.000**		
**positive**	**110**	**(34)**	**4.082**	**(1.980**–**6.993)**	**<0.0001**
**Stage: TNM class**					
**≤IIA**	**228**	**(71)**	**1.000**		
**IIB≤**	**92**	**(29)**	**3.704**	**(2.008**–**6.803)**	**<0.0001**
**ER**					
**positive**	**187**	**(58)**	**1.000**		
**negative**	**136**	**(42)**	**3.206**	**(1.694**–**6.070)**	**0.0003**
**PR**					
**positive**	**170**	**(53)**	**1.000**		
**negative**	**153**	**(47)**	**6.562**	**(2.919**–**14.751)**	**<0.0001**
HER2					
negative	290	(90)	1.000		
positive	33	(10)	2.151	(0.997–4.630)	0.0507
**Triple Negative**					
**others**	**236**	**(73)**	**1.000**		
**triple negative**	**87**	**(27)**	**4.149**	**(2.271**–**7.579)**	**<0.0001**
p53					
normal	243	(75)	1.000		
abnormal	80	(25)	1.737	(0.928–3.254)	0.0843
**RB1**					
**positive**	**307**	**(95)**	**1.000**		
**negative**	**16**	**(5)**	**5.325**	**(2.353**–**12.049)**	**<0.0001**
**RB1CC1**					
**positive**	**224**	**(69)**	**1.000**		
**negative**	**99**	**(31)**	**3.373**	**(1.840**–**6.185)**	**<0.0001**
**RB1CC1/RB1/p53**					
**normal**	**168**	**(52)**	**1.000**		
**abnormal**	**155**	**(48)**	**7.385**	**(3.116**–**6.185)**	**<0.0001**

The Cox proportional hazard regression model was used to evaluate the effects of clinico- pathological parameters on disease-specific-survival (DSS) with 95% confidence interval (95% CI). p-value <0.05, statistically significant. DSS intervals were used as the indicator for the relative-hazards.

### The combined evaluation of RB1CC1, RB1 and p53 provides the most significant prognostic prediction in Japanese breast cancer patients

**Figure 2:**
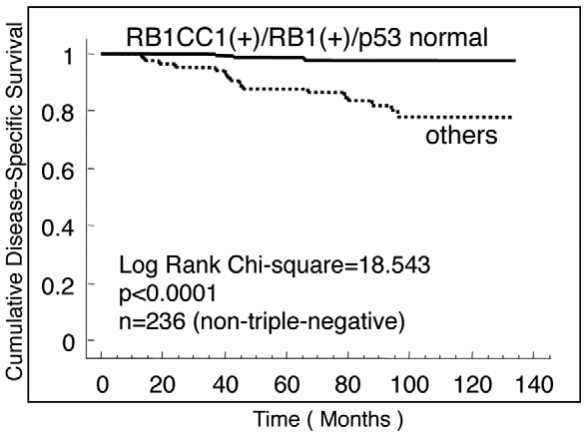
The combined evaluation of RB1CC1, RB1 and p53 provide a significant prediction of prognoses in non-triple-negative breast cancers. Kaplan-Meier method with a log-rank test was performed in 236 patients with non-triple-negative breast cancers (Chi-Square value = 18.543, p<0.0001).

To confirm the status of RB1, RB1CC1 and p53 as prognostic indicators of breast cancer, their expressions in breast cancer tissues of a larger cohort of 323 Japanese patients were immunohistochemically evaluated, and the correlation with the clinical data was analyzed statistically. Sixteen cases lacking RB1 expression had poor prognosis (Table 2; Fig. S2). RB1CC1 (−) status and p53ab were present in 99 and 80 cases, respectively, in this larger cohort. RB1CC1 (−) status was associated with the worst prognosis for DSS in this series (Log-rank test; Chi-Square value = 17.462, p<0.0001; Fig. 1A), quite similar to the results associated with RB1 (−) cases. p53 status alone had no statistically significant correlation with DSS (Log-rank test; Chi-Square value = 3.059, p = 0.0803; Fig. 1B). It is important to note that the prognosis of 168 cases without any deficit in RB1CC1/RB1/p53 immunoreactivity was significantly better than that of 155 cases with deficits in any one of these three components (Log-rank test; Chi-Square value = 28.496, p<0.0001), and that very few increments in breast cancer-specific death in the former group occurred even after five years from clinical disease onset (Fig. 1C). In this series, DSS of triple-negative breast cancers became distinctly worse than that of the remaining cases year by year for 3–5 years (Log-rank test; Chi-Square value = 25.279, p<0.0001), but the difference gradually decreased thereafter (Fig. 1D). A multivariate Cox proportional hazards analysis showed that RB1CC1 (−) status was a statistically significant risk for DSS (Hazard ratio = 2.037, p = 0.0310) in addition to the risks of triple-negative, TNM high-class, and chemotherapy-performed status (Fig. S3A). More important, dysfunction of either one of RB1CC1, RB1, or p53 was associated with the highest risk for disease-specific death (Multivariate Cox proportional hazards ratio = 3.951, 95% Confidence Interval = 1.566–9.967, p = 0.0036; Table 3). In addition, the combined evaluation of RB1CC1, RB1, and p53 predicted a longer DSS in the 236 cases with non-triple-negative cancers (Log-rank test, Chi-Square value = 18.543, p<0.0001; Fig. 2); i.e., no cancer-specific deaths were recorded among RB1CC1(+)/RB1(+)/p53nor patients at the follow-up after more than 5 years. With regard to disease-free survival (DFS), RB1CC1 was a risk factor (Log-rank test, Chi-Square value = 13.419, p = 0.0002; Fig. S4A). The combined evaluation of RB1CC1, RB1 and p53 also provided more accurate information than simple evaluation of p53 or triple-negativity [ER(−), PR(−) and HER2(−)] (Log-rank test, Chi-Square value = 19.295, p<0.0001; Fig. S4A–D). However, a multivariate Cox proportional hazards analysis indicated that DFS risk was not associated with the status of RB1CC1/RB1/p53 or triple-negativity, but with TNM high-class and chemotherapy-performed status (Fig. S4E).

**Table 3:** Multivariate Cox proportional hazards analysis for molecular markers and clinicopathological parameters.

	Disease-Specific Survival		
Covariate (n = 323)	hazard ratio	(95% CI)	p -values
**RB1CC1/RB1/p53 IHC (normal vs abnormal)**	**3.951**	**1.566**–**9.967**	**0.0036**
**Subtype ER/PR/HER2 (others vs triple negative)**	**1.974**	**1.038**–**3.754**	**0.0382**
**Stage: TNM class (≤IIA vs IIB≤)**	**2.188**	**1.149**–**4.167**	**0.0171**
**Chemotherapy (none vs performed)**	**2.242**	**1.012**–**4.950**	**0.0465**

The Cox's model was used to evaluate any independent prognostic effect of the variables on disease-specific survival (DSS) with a 95% confidence interval (95% CI). p-value <0.05, statistically significant. DSS intervals were used as the indicator for the relative-hazards.

## Discussion

Nuclear expression of RB1CC1 could be important for tumor suppression. As reported previously, RB1CC1 is located not only in the nuclei but also in the cytoplasm[11], [16], [18], [19]. Cytoplasmic RB1CC1 has been suggested as a possible equivalent of yeast Atg17, and several studies have indicated that RB1CC1 functions as an essential molecule in autophagy regulation[16], [17], [18]. Autophagy has been implicated in tumorigenesis[21], [22], but its precise role is ambiguous[23]. It is conceivable that autophagy has different roles in the different stages, or contexts, of tumorigenesis[23], [24], [25], [26], [27]. Young, et al.[28] have reported that autophagy mediates the mitotic senescence, an early window into tumor development. We suggest that cytoplasmic-nuclear transition of RB1CC1 plays a key role in the autophagy-senescence association. Cytoplasmic RB1CC1 seems to play no role as a direct tumor suppressor[20]. In fact, Martin, et al.[29] have reported that PIASy (a protein-inhibitor of activated STAT protein y) interacts with RB1CC1 and recruits an interacting complex between PIASy and RB1CC1 from cytoplasm into nuclei. In nuclei, PIASy positively activates the p53-p21 signaling pathway together with nuclear RB1CC1. Our recent data[20] demonstrated that nuclear RB1CC1 forms a large transcriptional complex with hSNF5, p53 and/or PIASy that activates a global transcription of genes (*RB1, p16* and *p21*) involved in the RB1 pathway—indicating a possible linkage to mitotic senescence—and suppresses tumor cell growth. Therefore, a positive status of nuclear RB1CC1 expression [RB1CC1(+)] appears to be intimately related to tumor suppression in breast cancer.

Evaluations of ER, PR, and HER2 in tumor tissue are useful for predicting the potential outcome of postoperative adjuvant therapy of breast cancer; thus it was demonstrated that patients with triple-negative cancers had an obviously worse outcome than non-triple-negative cases during shorter follow-up periods of up to 3–5 years[1], [2], [3]. However, the ability of triple-negative status to predict the prognosis diminished considerably after more than 5 years, and had disappeared at 10 years[1], [2], [3], so another diagnostic tool to predict the prognosis, especially related to DSS lasting more than 5 years, is needed. RB1CC1 is a novel regulator of RB1 that dephosphorylates RB1[8], [11] and increases its expression[8], [9], [19]. In addition, the RB1CC1-RB pathway plays an important role in the proliferation of breast cancer cells *in vitro* [20], and its genetic rearrangement has been demonstrated in breast cancer tissue *in vivo* [9], [10]. Accordingly, RB1CC1 itself and/or molecules (such as p53, SNF5 and PIASy) involved in the RB1CC1-RB1 pathways may be effective biomarkers to evaluate the clinical status of breast cancer patients.

In this report, using the hospital-based cohort of 323 breast cancer cases in Japan, we have shown that RB1CC1 status predicts breast cancer-specific survival (DSS). It is important to note that other established risk factors, such as chemotherapy, tumor size, lymph node status, TNM classification, ER, PR, triple-negative phenotype, and RB1 also conferred significant univariate relative hazards for DSS, thus confirming that the present cohort was a representative population. This population was not selected for RB1CC1 status, and is thus suitable to provide an unbiased assessment of RB1CC1 as a prognostic factor. In this cohort, RB1CC1(−) status correlated significantly with PR-negative and triple-negative phenotypes, as well as chemotherapy, and these findings seem to be closely related because chemotherapy was often applied to the PR-negative and/or triple-negative breast cancer patients.

In this cohort, the combined evaluation of RB1CC1, RB1 and p53 predicted prognoses more accurately than that of nuclear RB1CC1 expression, especially related to DSS for more than 5 years. In this series, similar to the results found in previous reports[1], [2], [3], [30], [31], patients with non-triple-negative breast cancers had distinctly better survivals than did those with triple-negative cancers, but the difference between triple-negative and non-triple-negative cancers decreased at the longer follow-up. The disease-specific death of non-triple-negative breast cancer patients in later follow-up periods (>5 years) is one of the greatest clinical problems. The combined evaluation of RB1CC1, RB1 and p53 predicted a longer DSS after more than 5 years in the cases with non-triple-negative cancers; i.e., no cancer-specific death was recorded among RB1CC1(+)/RB1(+)/p53nor patients in the later follow-up periods (>5 years). This combined evaluation of RB1CC1/RB1/p53 can provide a benefit for the clinical management of breast cancer patients, and improve upon the individual evaluations of RB1, p53 or triple-negativity [ER(−), PR(−) and HER2(−)] to predict the long-term prognosis of breast cancer patients[1], [2], [3], [6], [30], [31], [32], [33]. Although precise genetic analyses of *RB1CC1, RB1* and *p53* may offer better information on the prognosis, these analyses are not widely available as routine clinical examinations because of the cumbersome and expensive methodologies involved. The immunohistochemical technique used in the present study provides a suitable performance with regard to time and cost, and sufficient accuracy for the clinical prediction of survival, especially for the longer DSS. This technique will also provide a way to keep each breast cancer patient under suitable medical surveillance for specific periods.

Taken together, we have established RB1CC1 as a novel prognostic predictor in a cohort of Japanese breast cancer patients. Further studies of larger population cohorts (with more than 1,000 patients and of different races) are expected to confirm the validity of the RB1CC1/RB1/p53 combination in predicting long-term prognoses of breast cancer patients.

## Materials and Methods

### Patients and histology

In the preliminary study, a small cohort of 58 breast cancer cases treated at Shiga University of Medical Science in 1999 was analyzed. Absence of nuclear RB1CC1 expression [RB1CC1 (−)] was associated with the worst prognosis for breast cancer-specific survival (DSS; Log-rank test, Chi-Square value = 11.151, p = 0.0008; Fig. S1A). In addition, cases with any dysfunction in RB1CC1/RB1/p53 had a DSS prognosis significantly worse than those without any deficit in these three components (Log-rank test; Chi-Square value = 13.699, p = 0.0002; Fig. S1B). These data suggest that the prognoses of breast cancer patients are predictable by the immunohistochemical status of RB1CC1, RB1 and p53 in the primary tumor specimens. Therefore, we analyzed the correlation between prognosis and the immunohistochemical findings of the molecules involved in the RB1CC1-RB pathway in a total of 381 consecutive patients with operable primary breast cancers treated at Shiga University of Medical Science (72 cases), or at Osaka Medical Center for Cancer and Cardiovascular Diseases (309 cases) between 1999 and 2000. Among these cases, we failed to perform immunohistochemical evaluations in 57 tumor specimens as a result of tissue loss during slide preparation. Specimens from 324 patients with operable primary breast cancer in the cohort were immunohistochemically evaluated, but post-operative clinical data were not available in one case. Therefore, 323 cases were available for the statistical analysis of immunohistochemical and clinical data in this study. Data were collected from clinical and pathological records with the written informed consent of patients and after approval by the Ethics Committee of each institution; Shiga University of Medical Science and Osaka Medical Center for Cancer and Cardiovascular Diseases. The pathological diagnoses of all the specimens were confirmed by at least two surgical pathologists (Y.T. and H.O.), and classified according to the World Health Organization (WHO) guidelines. In the present cohort, the mean follow-up period was 78.4 months (standard error  = 1.70 months; range 3–134 months). The mean age at diagnosis was 53.4 years (standard error  = 0.65 years; range 26–90 years).

### Antibodies and reagents

Rabbit antisera against RB1CC1 (aa. 25–271, 549–817 as each epitope) were generated as previously reported[13], [19], and the purified IgGs were used in the experiments. Anti-p53 (FL-393) and p21 (F-5) were obtained from Santa Cruz Biotechnology (CA). Anti-p53 (DO-7), ER (1D5) and PR (PgR636) were purchased from Sigma (MO). Anti-HER2 (HercepTest) was from DAKO (Denmark). Anti-RB1 (G3–245) was from BD Biosciences (CA).

### Immunohistochemistry

Surgical specimens were transferred to 10% buffered formalin and fixed overnight. The fixed samples were embedded in paraffin and serially sliced into 5-µm sections. To evaluate RB1CC1, RB1, p53, p21, ER, PR and HER2 in human breast cancer tissues, deparaffinized sections were autoclaved at 120°C for 1 min, immersed in 0.3% H_2_O_2_ and rinsed with 1xPBS before incubation overnight at 4°C with each of the primary antibodies. The sections were rinsed with 1xPBS and incubated with the secondary antibody (Simple Stain MAX-PO; Nichirei, Japan) at room temperature for 1 hour. The sections were then stained with 3,3′-diaminobenzidine tetrahydrochloride (DAB), and counter-stained with hematoxylin.

### Microscopic evaluation and statistical analysis for clinical outcomes

The immunohistochemical results for RB1CC1 were first quantitatively graded as follows: I, negative stain in both cytoplasm and nuclei; II, positive stain only in cytoplasm and negative in nuclei; III, positive stain in nuclei with or without stain in cytoplasm. Our recent study proved that RB1 expression was significantly higher in cases with nuclear RB1CC1 expression (grade III) than in cases without nuclear RB1CC1 (grade I and II)[19]. In addition, we have found that nuclear RB1CC1 complexed with p53 and hSNF5 played a functional transcriptional role in the RB1 pathway and suppressed tumor cell growth[20]. Therefore, in the analysis of the present clinical cohort, RB1CC1 staining grades I-II and III were defined as -negative (−) and -positive (+), respectively; i.e., only the cases with nuclear RB1CC1 expression were recognized as RB1CC1 (+). A dysfunctional status of p53 was assessed immunohistochemically by the percentage of cells that were positive for p53 and p21. Similar to the assignments in the previous report[20], an abnormal p53 status (p53ab) was defined as a case of breast cancer if more than 50% of tumor cells were strongly positive for p53, while less than 10% of the cells were positive for p21. Cells were considered positive for ER and PR when more than 10% of the cells stained positive. According to *the criteria of the American Cancer Association*, HER2 expression was scored from 0 to 3+, and a 3+ score was defined as HER2-positive.

Disease-specific survival (DSS: breast cancer-specific survival) interval was defined by the period from clinical onset to death due to breast cancer, and disease-free survival (DFS) was the period until reappearance of breast cancer-related adverse events (i.e., breast cancer recurrence or distant metastasis). Statistical analysis was performed in StatView 5.0 for Windows (StatView Inc., NC). All tests for statistical significance were two-sided. A p-value of <0.05 was considered statistically significant. Bivariate analyses of the association between covariables and RB1CC1 status included Chi-square and Fisher's exact tests. Survival curves were estimated by the Kaplan-Meier method with a log-rank test and the Chi-square test to assess significance. The univariate and multivariate Cox proportional hazards regression models were used to evaluate any independent prognostic effect of the variables with a 95% confidence interval.

## Supporting Information

Figure S1 RB1CC1 is a predictive biomarker for breast cancer patients. In the preliminary cohort of 58 breast cancer cases, Kaplan-Meier survival curves with Log-rank tests indicated that (**A**) RB1CC1 was a significant predictor for disease-specific survival (DSS; Log-rank test, Chi-Square value = 11.151, p = 0.0008). (**B**) The combined evaluation of RB1CC1, RB1 and p53 was significantly correlated with DSS (Log-rank test, Chi-Square value = 13.699, p = 0.0002).
(TIF)

Figure S2 Sixteen cases lacking RB1 expression had poor prognosis. Kaplan-Meier survival curves were constructed from 16 cases with RB1-null status in the larger cohort of 324 breast cancer cases. (**A**) Disease-specific survival (DSS). (**B**) Disease-free survival (DFS).
(TIF)

Figure S3 RB1CC1 is an independent prognostic biomarker for DSS of breast cancer patients. (**A**) Multivariate Cox proportional hazards analysis showed that RB1CC1 (−) was a statistically significant risk for breast cancer-specific death (Hazard ratio = 2.037, 95% Confidence Interval = 1.067–3.887, p = 0.0310) in addition to the risks of triple-negative, TNM high-class, and chemotherapy-performed status in the 323 breast cancer patient cohort. (**B**) Multivariate Cox proportional hazards analysis for DFS in the cohort.
(TIF)

Figure S4 RB1CC1, RB1 and p53 status predicts clinical outcomes in cases of breast cancer. Kaplan-Meier survival curves with Log-rank tests were performed for DFS evaluation of (**A**) RB1CC1, (**B**) p53, (**C**) the combination of RB1CC1/RB1/p53, and (**D**) triple-negative for ER/PR/HER2. (**E**) Multivariate Cox proportional hazards analysis indicated that DFS risk was not associated with the status of RB1CC1/RB1/p53 or triple-negative, but with TNM high-class and chemotherapy-performed in the 323 Japanese breast cancer patient cohort.
(TIF)
