# Supplementary figures and images for: RB1CC1 Together with RB1 and p53 Predicts Long-Term Survival in Japanese Breast Cancer Patients
Source: PLoS One. 2010 Dec 22;5(12):e15737. doi: 10.1371/journal.pone.0015737 (PMC3008740; doi:10.1371/journal.pone.0015737)

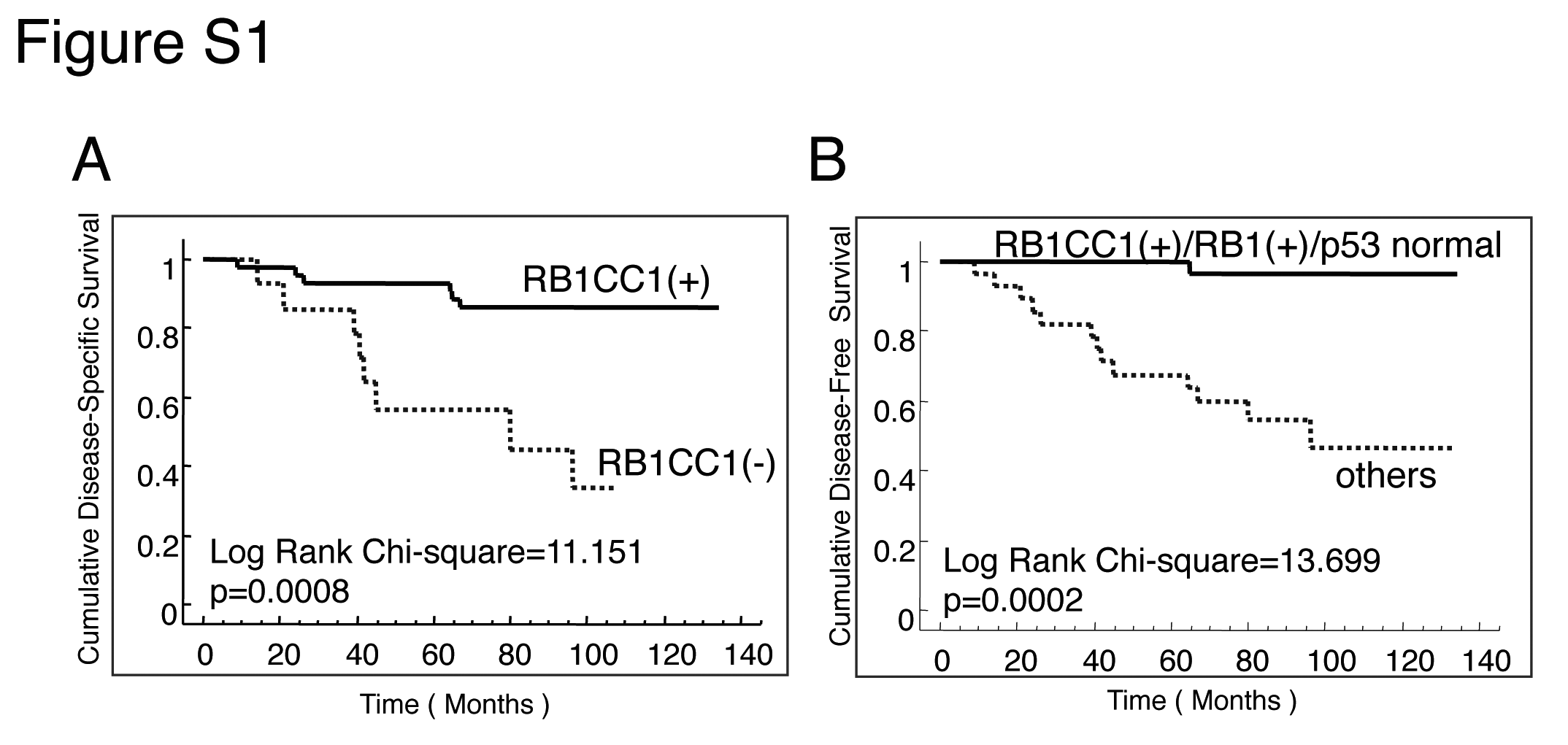

Supplement: Figure S1 — RB1CC1 is a predictive biomarker for breast cancer patients. In the preliminary cohort of 58 breast cancer cases, Kaplan-Meier survival curves with Log-rank tests indicated that (A) RB1CC1 was a significant predictor for disease-specific survival (DSS; Log-rank test, Chi-Square value = 11.151, p = 0.0008). (B) The combined evaluation of RB1CC1, RB1 and p53 was significantly correlated with DSS (Log-rank test, Chi-Square value = 13.699, p = 0.0002). (TIF) [file pone.0015737.s001.tif]

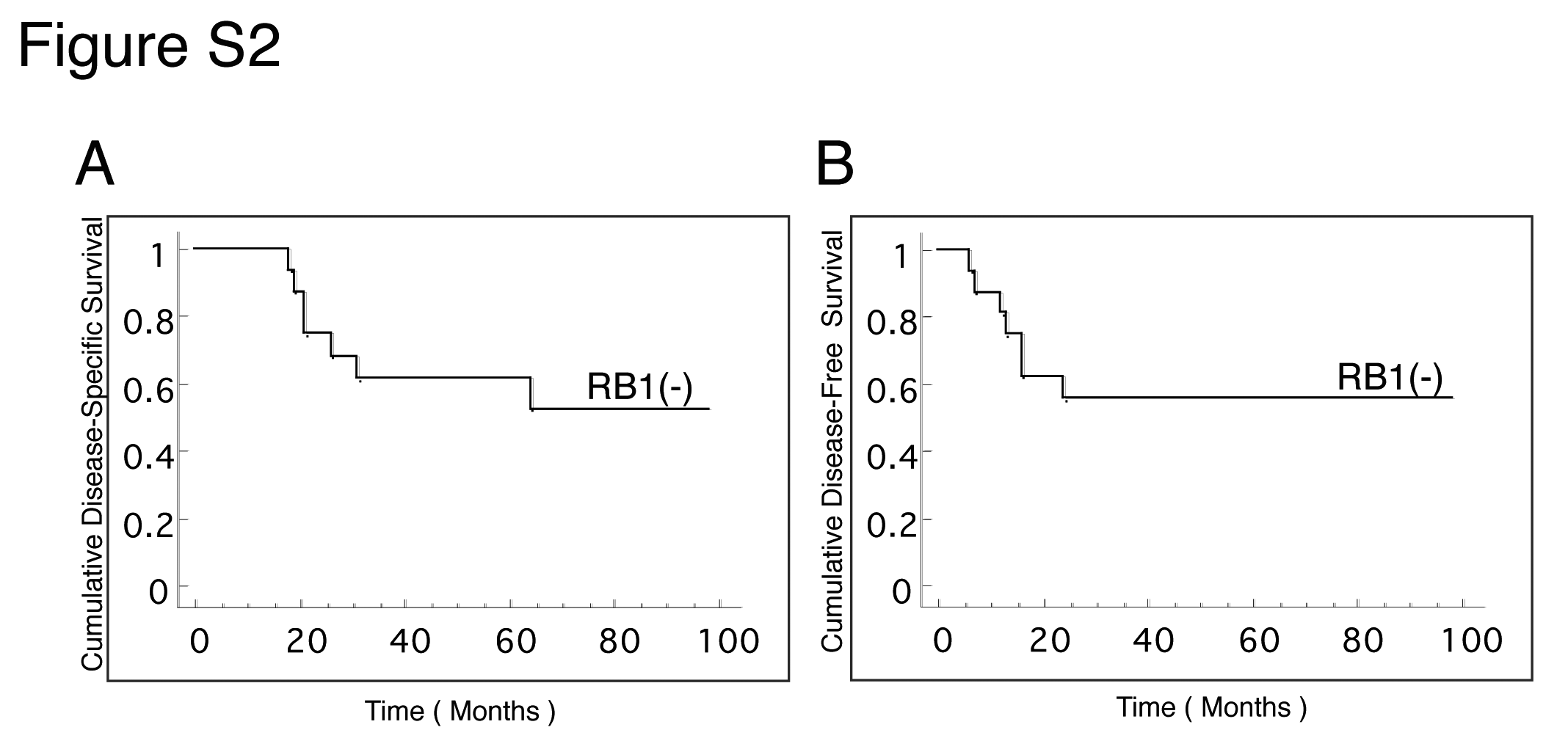

Supplement: Figure S2 — Sixteen cases lacking RB1 expression had poor prognosis. Kaplan-Meier survival curves were constructed from 16 cases with RB1-null status in the larger cohort of 324 breast cancer cases. (A) Disease-specific survival (DSS). (B) Disease-free survival (DFS). (TIF) [file pone.0015737.s002.tif]

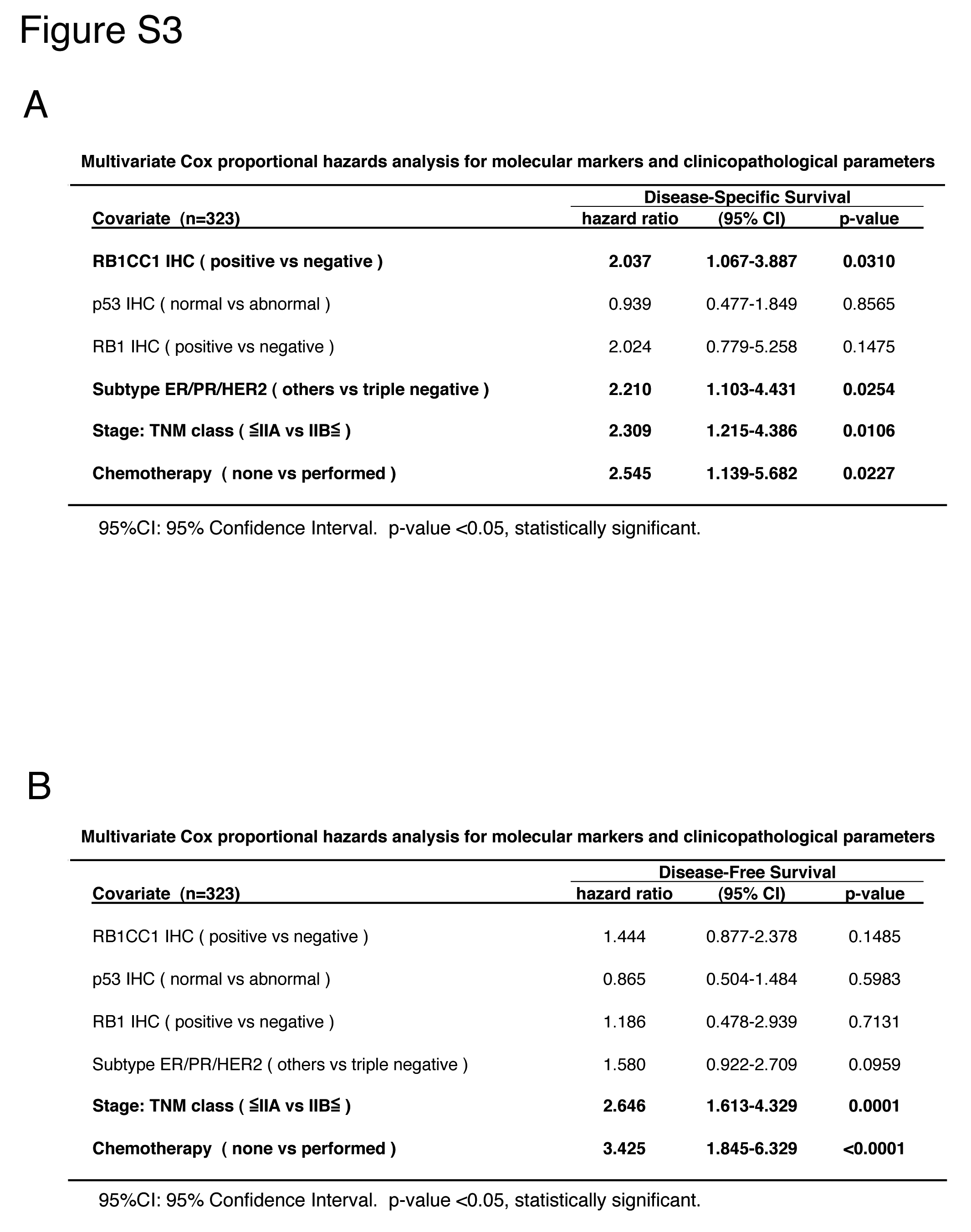

Supplement: Figure S3 — RB1CC1 is an independent prognostic biomarker for DSS of breast cancer patients. (A) Multivariate Cox proportional hazards analysis showed that RB1CC1 (−) was a statistically significant risk for breast cancer-specific death (Hazard ratio = 2.037, 95% Confidence Interval = 1.067–3.887, p = 0.0310) in addition to the risks of triple-negative, TNM high-class, and chemotherapy-performed status in the 323 breast cancer patient cohort. (B) Multivariate Cox proportional hazards analysis for DFS in the cohort. (TIF) [file pone.0015737.s003.tif]

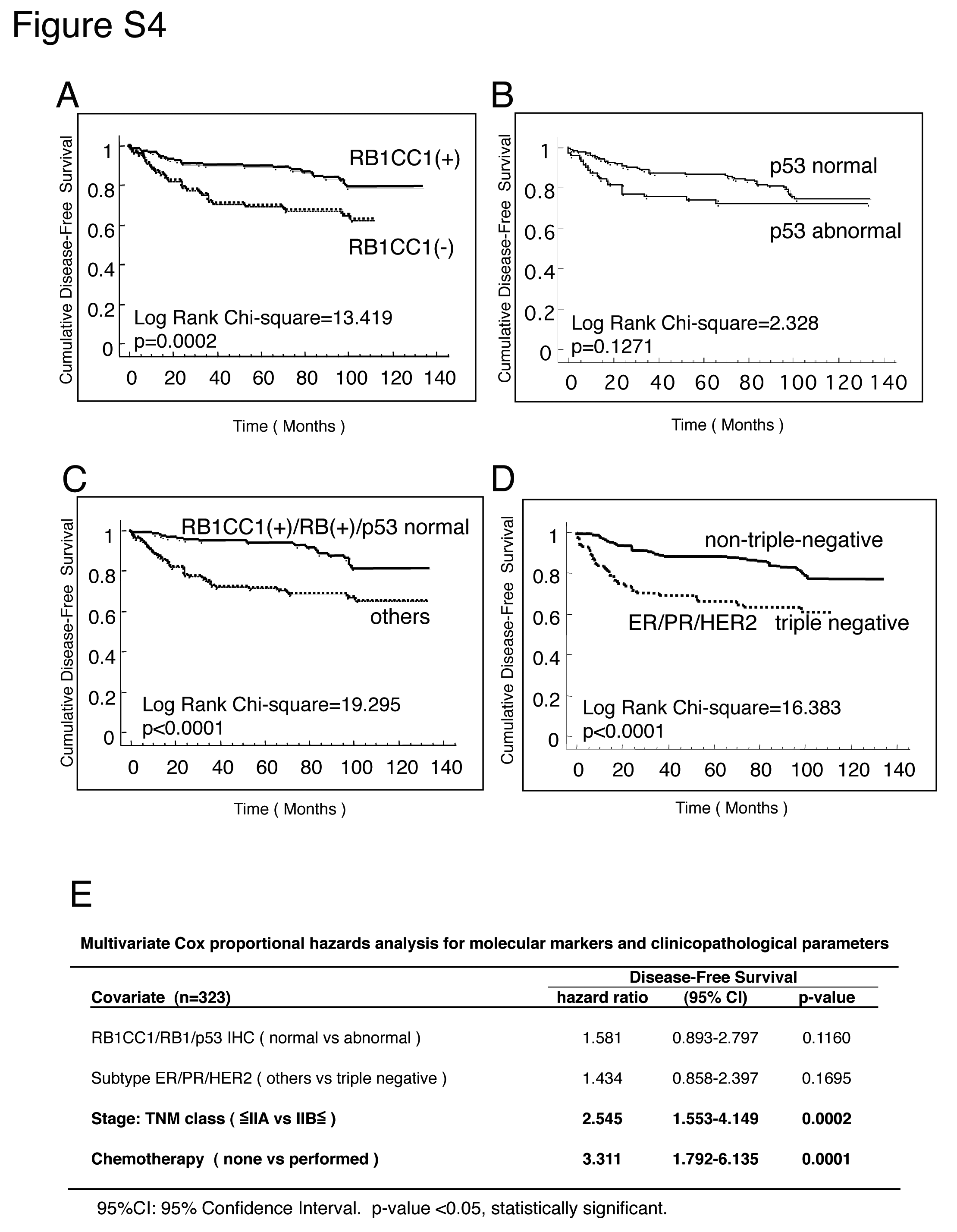

Supplement: Figure S4 — RB1CC1, RB1 and p53 status predicts clinical outcomes in cases of breast cancer. Kaplan-Meier survival curves with Log-rank tests were performed for DFS evaluation of (A) RB1CC1, (B) p53, (C) the combination of RB1CC1/RB1/p53, and (D) triple-negative for ER/PR/HER2. (E) Multivariate Cox proportional hazards analysis indicated that DFS risk was not associated with the status of RB1CC1/RB1/p53 or triple-negative, but with TNM high-class and chemotherapy-performed in the 323 Japanese breast cancer patient cohort. (TIF) [file pone.0015737.s004.tif]
